# rAAV-mediated overexpression of TGF-β stably restructures human osteoarthritic articular cartilage *in situ*

**DOI:** 10.1186/1479-5876-11-211

**Published:** 2013-09-13

**Authors:** Jagadeesh K Venkatesan, Ana Rey-Rico, Gertrud Schmitt, Anna Wezel, Henning Madry, Magali Cucchiarini

**Affiliations:** 1 Center of Experimental Orthopaedics, Saarland University Medical Center, Kirrbergerstr. Bldg 37, Homburg/Saar 66421, Germany; 2 Department of Orthopaedic Surgery, Saarland University Medical Center, Homburg/Saar, Germany

**Keywords:** Human osteoarthritis, Articular cartilage, rAAV gene transfer, TGF-β

## Abstract

**Background:**

Therapeutic gene transfer is of significant value to elaborate efficient, durable treatments against human osteoarthritis (OA), a slow, progressive, and irreversible disorder for which there is no cure to date.

**Methods:**

Here, we directly applied a recombinant adeno-associated virus (rAAV) vector carrying a human transforming growth factor beta (TGF-β) gene sequence to primary human normal and OA chondrocytes *in vitro* and cartilage explants *in situ* to monitor the stability of transgene expression and the effects of the candidate pleiotropic factor upon the regenerative cellular activities over time.

**Results:**

Efficient, prolonged expression of TGF-β achieved via rAAV gene transfer enhanced both the proliferative, survival, and anabolic activities of cells over extended periods of time in all the systems evaluated (at least for 21 days *in vitro* and for up to 90 days *in situ*) compared with control (reporter) vector delivery, especially *in situ* where rAAV-hTGF-β allowed for a durable remodeling of OA cartilage. Notably, sustained rAAV production of TGF-β in OA cartilage advantageously reduced the expression of key OA-associated markers of chondrocyte hypertrophic and terminal differentiation (type-X collagen, MMP-13, PTHrP, β-catenin) while increasing that of protective TIMPs and of the TGF-β receptor I in a manner that restored a favorable ALK1/ALK5 balance. Of note, the levels of activities in TGF-β-treated OA cartilage were higher than those of normal cartilage, suggesting that further optimization of the candidate treatment (dose, duration, localization, presence of modulating co-factors) will most likely be necessary to reproduce an original cartilage surface in relevant models of experimental OA *in vivo* without triggering potentially adverse effects.

**Conclusions:**

The present findings show the ability of rAAV-mediated TGF-β gene transfer to directly remodel human OA cartilage by activating the biological, reparative activities and by regulating hypertrophy and terminal differentiation in damaged chondrocytes as a potential treatment for OA or for other disorders of the cartilage that may require transplantation of engineered cells.

## Introduction

Osteoarthritis (OA) is a major, widespread degenerative disease of the entire joint characterized by complex structural and functional tissue and cell alterations [1-5] for which there is no cure to date. OA has a multifactorial etiology, being influenced by both genetic, mechanical, and environmental factors [6-8]. The gradual and irreversible degradation of the articular cartilage in OA, associated with a remodeling of the subchondral bone and osteophyte formation, is the result of an impaired cartilage homeostasis (prevalence of catabolic events activated by biomechanical and pro-inflammatory mediators, failure of the chondrocytes to preserve and restore the metabolic balance) [9,10]. Thus far, none of the pharmacological treatments and surgical options available to manage OA have allowed to reproduce the original cartilage integrity in patients. The design of new therapeutic approaches for OA is therefore of crucial importance to effectively and durably counteract the regular progression of the disease by activating regenerative processes in the chondrocytes as a means to re-equilibrate the disturbed cartilage balance.

Therapeutic gene transfer is a valuable tool to achieve this goal as it has the potential to allow for the production of factors over extended periods of time compared with the application of recombinant molecules with short pharmacological half-lives. While protection against cartilage breakdown was afforded by delivering sequences coding for agents with preventive and/or inhibitory activities (an IL-1 receptor antagonist - IL-1Ra, siRNAs against IL-1 or ADAMTS-5, soluble IL-1 and TNF receptors - sIL-1R and sTNFR, NF-κB inhibitors, kallistatin - KBP, thrombospontin-1 - TSP-1, Dickkopf-1 - DKK-1, pro-opiomelanocortin - POMC) [11-21], compensation for the loss of matrix elements and cells was not achieved to further re-establish an original cartilage surface in these various experimental systems. Instead, such effects have been ascribed, at least to some extent, to gene transfer of factors with anabolic and/or proliferative properties like proteoglycan 4 [22], the insulin-like growth factor I (IGF-I) [18,23,24], fibroblast growth factor 2 (FGF-2) [25,26], bone morphogenetic proteins 2 and 4 (BMP-2, -4) [23,27], and the transcription factor SOX9 [28,29].

Yet, even in the presence of such agents, only partial cartilage resurfacing was noted, showing the need to identify other components of therapeutic value for improved gene transfer applications in OA. Equally important, the development of an effective treatment for OA will necessitate that the gene vehicle promotes the stable expression of a candidate sequence that can durably counteracts the slow and irreversible progression of the disease. In this regard, the transforming growth factor beta (TGF-β) is an attractive candidate owing to its prominent, pleiotropic effects upon cartilage formation, chondrocyte proliferation, and extracellular matrix (ECM) synthesis and to its ability to suppress IL-1-induced cartilage breakdown [30-33]. Yet, little is known on the effects of TGF-β gene transfer and overexpression in primary human OA articular chondrocytes and articular cartilage over relevant, extended periods of time. Most remarkably, Ulrich-Vinther *et al*. [34] reported that delivery of TGF-β via the promising recombinant adeno-associated virus (rAAV) vectors resulted in increased levels of type-II collagen and aggrecan and reduced expression of matrix metalloproteinase 3 (MMP-3) in human OA chondrocytes *in vitro* for about a week although effects at later time points were not documented. As a matter of fact, rAAV are among the most advantageous classes of vectors available for therapy to date, especially for use as a gene transfer system in OA. rAAV derived from a human non-pathogenic replication-defective virus carry no viral coding sequences in the recombinant genome, making them less immunogenic than adenoviral vectors [23,35,36]. rAAV can modify the quiescent chondrocytes both *in vitro* and *in situ* in their dense ECM at very high efficiencies and for prolonged periods of time, probably due to their small size (20 nm) and to a good maintenance of the constructs in the host under episomal forms [24,26,28,34,37,38]. This is in marked contrast with nonviral [39] and adenoviral vectors [23,35,36] that mediate only short-term transgene expression, and with retroviral vectors [40,41] that require cell division and selection and carry the risk of insertional mutagenesis following integration in the host genome.

In the present study, we tested whether efficient TGF-β overexpression can be achieved over prolonged periods of time via rAAV gene transfer in primary chondrocytes and explant cultures prepared from the articular cartilage of normal donors and OA patients (the ultimate targets for therapy), leading to enhanced levels of cell proliferation, survival, and matrix synthesis compared with control (reporter gene vector) treatment. We further analyzed the extent by which the candidate rAAV TGF-β treatment is capable of restructuring OA cartilage compared with normal (control) cartilage and explored the pathways potentially implicated in the remodeling processes.

## Materials and methods

### Reagents

All reagents were from Sigma (Munich, Germany) except for the dimethylmethylene blue (DMMB) dye (Serva, Heidelberg, Germany). The anti-TGF-β (V), anti-MMP-13 (72B-01), anti-TIMP-1 (C-20) and -TIMP-3 (W-18), anti-parathyroid hormone-related protein (PTHrP) (1D1), anti-β-catenin (E-5), and anti-TGF-β receptor I (activin receptor-like kinase-1 ALK1: C-20; ALK5: T-19) antibodies were from Santa Cruz Biotechnology (Heidelberg, Germany). The anti-type-II collagen (AF-5710) was antibody from Acris (Hiddenhausen, Germany). The anti-type-X collagen (COL-10) and anti-BrdU (BU-33) antibodies were from Sigma. Active TGF-β secretion was monitored with the hTGF-β Quantikine ELISA (DB100B; R&D Systems; Wiesbaden, Germany). The Cell Proliferation ELISA BrdU was from Roche Applied Science (Mannheim, Germany). The ApopTag® Plus Peroxidase *In Situ* Apoptosis Detection Kit was from Chemicon-Millipore (Schwalbach/Ts., Germany). The type-II collagen contents were measured with the native type-II collagen Arthrogen-CIA Capture ELISA kit (Chondrex, Redmond, WA, USA) and those for type-X collagen using a COL-10 ELISA (Antibodies-Online, Aachen, Germany).

### Cartilage and cells

Human normal articular cartilage was obtained from unaffected knee joints removed during tumor surgery (n = 8, age 65–73). OA was excluded on safranin O-stained sections using the Mankin score [42] (score 1–2). OA cartilage was obtained from joints undergoing total knee arthroplasty (n = 14, age 65–78) (Mankin score 7–9). The study was approved by the Ethics Committee of the Saarland Physicians Council. Research has been performed in accordance with the Declaration of Helsinki involving human material. Informed consent has been obtained from all participants. Explant cultures and chondrocytes (passage 1–2) were prepared as previously described [24,26,28,38].

### Plasmids and rAAV vectors

rAAV-*lacZ* is an AAV-2-based plasmid [43,44] carrying the *lacZ* gene encoding β-galactosidase under the control of the cytomegalovirus immediate-early (CMV-IE) promoter [24,26,28,38]. rAAV-hTGF-β carries a 1.2-kb human transforming growth factor beta 1 (hTGF-β) cDNA fragment (intronless open reading frame from the ATG to the stop codon) (pORF9-hTGFB1) (Invivogen, Toulouse, France) that was cloned in rAAV-*lacZ* in place of *lac*Z (the fragment was confirmed by sequencing). rAAV were packaged as conventional (not self-complementary) vectors using a helper-free, two-plasmid transfection system in the 293 cell line (an adenovirus-transformed human embryonic kidney cell line) using the packaging plasmid pXX2 and the Adenovirus helper plasmid pXX6 as previously described [45]. Vector preparations were purified by dialysis and titered by real-time PCR (about 10^10^ transgene copies/ml, with a ratio viral particles-to-functional vector of 500/1) [24,26,28,38].

### Gene transfer

The vectors were applied to the samples based on concentrations previously tested [24,26,28]. Chondrocytes (2 × 10^4^) were transduced with rAAV (40 μl, i.e. 8 × 10^5^ functional recombinant viral particles; multiplicity of infection MOI = 40) and cultured for up to 21 days, while explant cultures were transduced by direct application of the vectors (40 μl) onto the surface of the samples and cultured for up to 90 days [24,26,28,38].

### Transgene expression

Transgene (TGF-β) expression was monitored by indirect immunostaining using a specific antibody, a biotinylated secondary antibody (Vector Laboratories), and the ABC method (Vector Laboratories) using diaminobenzidine (DAB) as the chromogen. Samples were examined under light microscopy (Olympus BX 45; Hamburg, Germany) [24,26,28,38]. Expression of TGF-β was also assayed by ELISA at the denoted time points (*in vitro*: days 5 and 21; *in situ*: days 21 and 90).

### Histological and immunohistochemical analyses

Cell and explant cultures were fixed and explants were processed to stain paraffin-embedded sections (5 μm) using safranin O to detect proteoglycans and hematoxylin eosin (H&E) to detect cells [24,26,28]. Expression of type-II and type-X collagen, MMP-13, TIMP-1 and −3, PTHrP, β-catenin, and the TGF-β receptor I (ALK1 and ALK5) was detected with specific antibodies, biotinylated secondary antibodies, and the ABC method with DAB. Samples were examined under light microscopy (Olympus BX 45).

### Cell proliferation and apoptosis assays

The proliferative activities were assessed by immunolabeling after BrdU incorporation [24]. Briefly, BrdU was introduced at a final concentration of 3 μg/ml in the culture medium 24 h after rAAV transduction. Samples were immunochemically processed to monitor the proliferation rates with a specific anti-BrdU antibody, a biotinylated secondary antibody, and the ABC method with DAB. Proliferation was also assessed using the Cell Proliferation ELISA BrdU, with OD proportional to the cell numbers, as previously described [46]. *In situ*, nuclear DNA fragmentation consistent with apoptosis was determined by the terminal deoxynucleotidyl transferase-mediated dUTP nick end labeling (TUNEL) method [24,26].

### Morphometric analyses

The transduction efficiencies (ratio of cells positive for TGF-β immunolabeling to the total number of cells on immunohistological sections), the cells positive for BrdU uptake (ratio of cells positive for BrdU immunolabeling to the total number of cells on immunohistological sections), the cell densities (cell numbers/mm^2^ of surface of the site evaluated on histological sections), the apoptotic cells (ratio of cells positive for TUNEL assay to the total number of cells on immunohistological sections), the safranin O staining intensities (ratio of tissue surface positively stained by safranin O to the total surface of the site evaluated on histological sections), the type-II or type-X collagen immunostaining intensities (ratio of tissue surface positively immunostained by type-II or type-X collagen to the total surface of the site evaluated on immunohistological sections), as well as the cells positive for the expression of MMP-13, TIMP-1 and −3, PTHrP, β-catenin, and the TGF-β receptor I (ALK1, ALK5, ALK1/ALK5 ratio) (ratio of cells positive for immunolabeling of each of these markers to the total number of cells on immunohistological sections) were measured at three random sites standardized for their surface or using ten serial histological and immunohistochemical sections for each parameter, test, and replicate condition to allow for calculation of standard deviations (SD). Analysis programs included SIS AnalySIS (Olympus), Adobe Photoshop (Adobe Systems, Unterschleissheim, Germany), and Scion Image (Scion Corporation, Frederick, MD, USA) [24,26,28].

### Biochemical assays

Explant cultures were processed for the assays as previously described [24,26,28]. The DNA contents were determined using Hoechst 33258, the proteoglycan contents by binding to the DMMB dye, and those for type-II collagen and type-X collagen by ELISA [24,26,28,47]. Data were normalized to total cellular proteins using a protein assay (Pierce Thermo Scientific, Fisher Scientific, Schwerte, Germany). All measurements were performed with a GENios spectrophotometer/fluorometer (Tecan, Crailsheim, Germany).

### Statistical analysis

Each condition was performed in triplicate in three independent experiments with both types of cultures. Data were obtained by two individuals that were blinded with respect to the treatment groups. Values are expressed as mean ± standard deviation (SD). The *t*-test and Mann–Whitney Rank Sum Test were employed where appropriate. *P* values of less than 0.05 were considered statistically significant.

## Results

### rAAV-mediated TGF-β overexpression in human normal and OA articular chondrocytes *in vitro* and *in situ*

The functionality of the rAAV-hTGF-β vector was first tested in human normal and OA primary chondrocyte cultures and articular cartilage explants.

**Figure 1:**
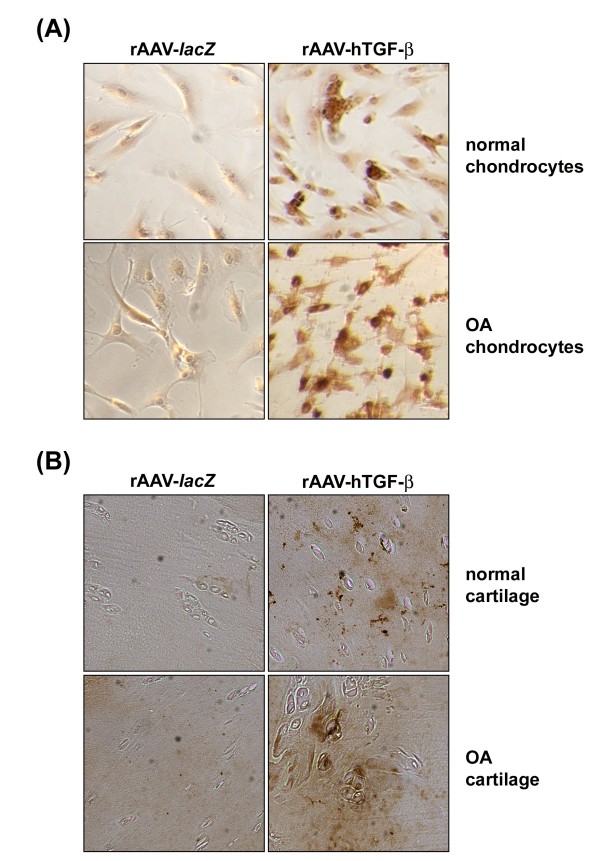
**Detection of TGF-β expression in rAAV-transduced human normal and OA chondrocytes in vitro and in situ.** Cells **(A)** and explants **(B)** were transduced by direct administration of the vectors (rAAV-lacZ or rAAV-hTGF-β: 40 μl each vector) and maintained in culture for 21 days in vitro and for 90 days in situ. The samples were then fixed and histologically processed to monitor the expression of TGF-β by immunocyto-/-histochemical detection **(A**: magnification x4; **B**: magnification x20, view of the middle zone**)**.

*In vitro*, significant, sustained (at least 21 days) TGF-β expression was noted only in rAAV-hTGF-β-transduced chondrocytes compared with the control (rAAV-*lacZ*) condition (normal cells: from 461.2 ± 7.8 to 184.2 ± 3.5 *versus* 14.6 ± 2.1 to 11.3 ± 0.9 pg/ml/24 h between days 5 and 21; OA cells: from 552.4 ± 6.5 to 219.4 ± 3.2 *versus* 17.5 ± 3.1 to 10.6 ± 0.7 pg/ml/24 h between days 5 and 21; up to 31.6-fold difference, always *P* ≤ 0.001), showing durable transduction efficiencies (up to 80%) (Figure 1A).

Significant, durable (at least 90 days) TGF-β expression was also achieved *in situ* when applying rAAV-hTGF-β to cartilage explants compared with rAAV-*lacZ* (normal cartilage: from 724.5 ± 4.9 to 304.2 ± 2.2 *versus* 92.3 ± 1.1 to 55.2 ± 1.9 pg/ml/24 h between days 21 and 90; OA cartilage: from 987.7 ± 4.8 to 324.9 ± 4.3 *versus* 83.4 ± 2.1 to 58.1 ± 3.2 pg/ml/24 h between days 21 and 90; up to 11.8-fold difference, always *P* ≤ 0.001), with specific immunoreactivity observed both in the superficial and middle zones of the cartilage and showing again durable transduction efficiencies (up to 70%) (Figure 1B).

These results show that the current rAAV TGF-β vector is capable of modifying human normal and OA chondrocytes both *in vitro* and *in situ*, allowing for significant levels of transgene expression compared with control vector administration over extended periods of time, especially when the cells are embedded in their ECM (at least 90 days *in situ*).

### Effects of rAAV-hTGF-β administration upon the cellular activities of human normal and OA articular chondrocytes *in vitro* and *in situ*

We next evaluated the ability of rAAV-mediated TGF-β overexpression to stimulate the proliferative and survival activities of chondrocytes in the systems tested above.

**Figure 2:**
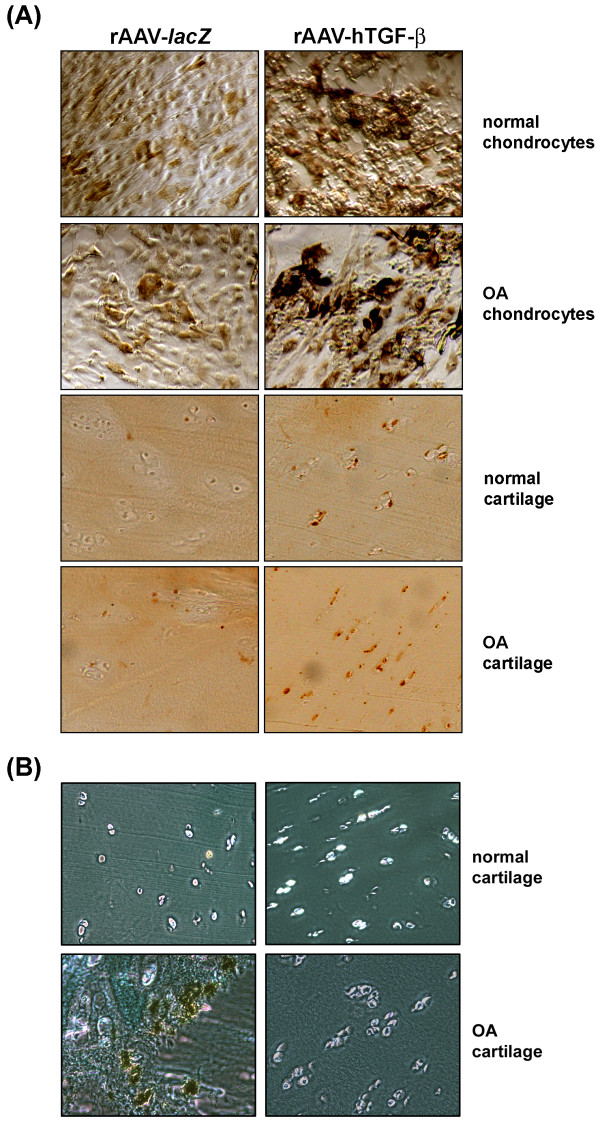
**Effects of rAAV-mediated TGF-β expression upon the proliferative and anti-apoptotic activities in human normal and OA chondrocytes *in vitro *and *in situ*.** Cells and explants were transduced with rAAV-*lacZ* or rAAV-hTGF-β as described in Figure 1 and maintained in culture for 21 days *in vitro* and for 90 days *in situ*. The samples were then fixed and histologically processed to detect the incorporation of BrdU by immunolabeling **(A)** (*in vitro*: magnification x2; *in situ*: magnification x10) and to monitor apoptotic events by TUNEL assay **(B)** (magnification x10). View of the middle zone.

**Figure 3:**
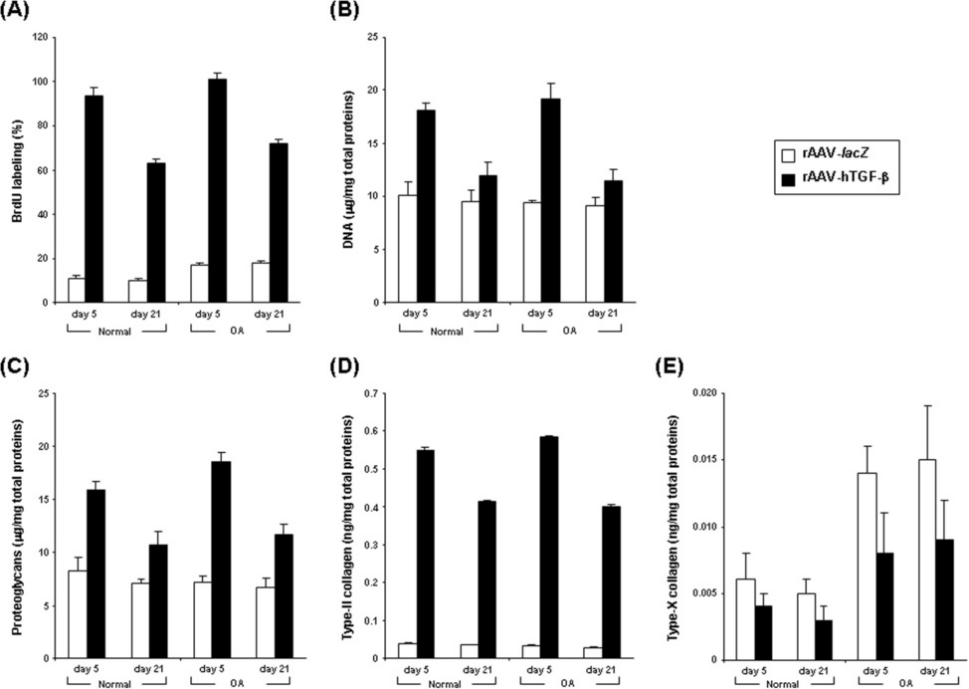
**Morphometric and biochemical analyses in rAAV-transduced chondrocytes *in vitro*.** Cells were transduced with rAAV-*lacZ* or rAAV-hTGF-β as described in Figure 1 and maintained in culture for up to 21 days. The samples were then fixed and histologically processed at the denoted time points to monitor the % of BrdU labeling **(A)** and the contents of DNA **(B)**, proteoglycans **(C)**, type-II **(D)** and type-X collagen **(E)**.

**Figure 4:**
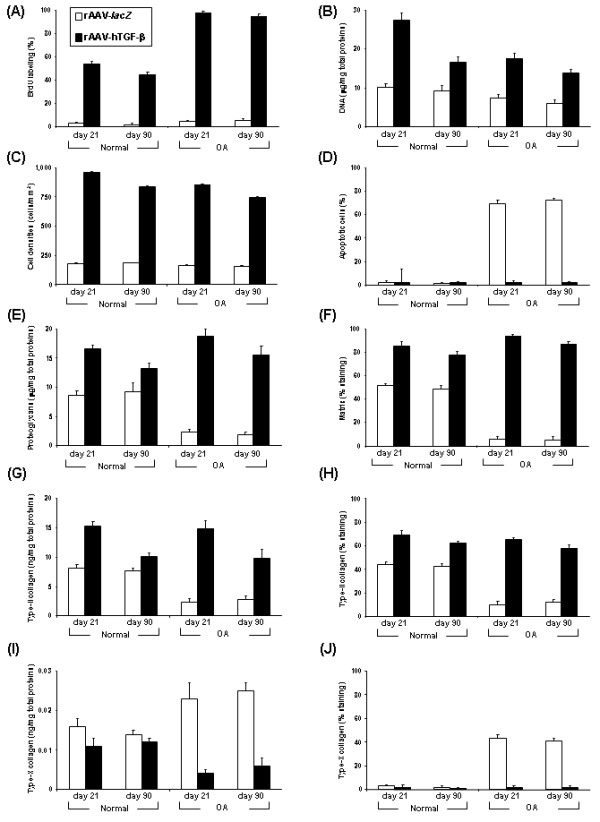
**Morphometric and biochemical analyses in rAAV-transduced chondrocytes *in situ*.** Explants were transduced with rAAV-*lacZ* or rAAV-hTGF-β as described in Figure 1 and maintained in culture for up to 90 days. The samples were then fixed and histologically processed at the denoted time points to monitor the % of BrdU labeling **(A)**, the DNA contents **(B)**, cells densities **(C)**, % of apoptotic cells **(D)**, proteoglycan contents **(E)**, % of matrix staining **(F)**, type-II collagen contents **(G)**, % of type-II collagen immunostaining **(H)**, type-X collagen contents **(I)**, and % of type-X collagen immunostaining **(J)**.

**Figure 5:**
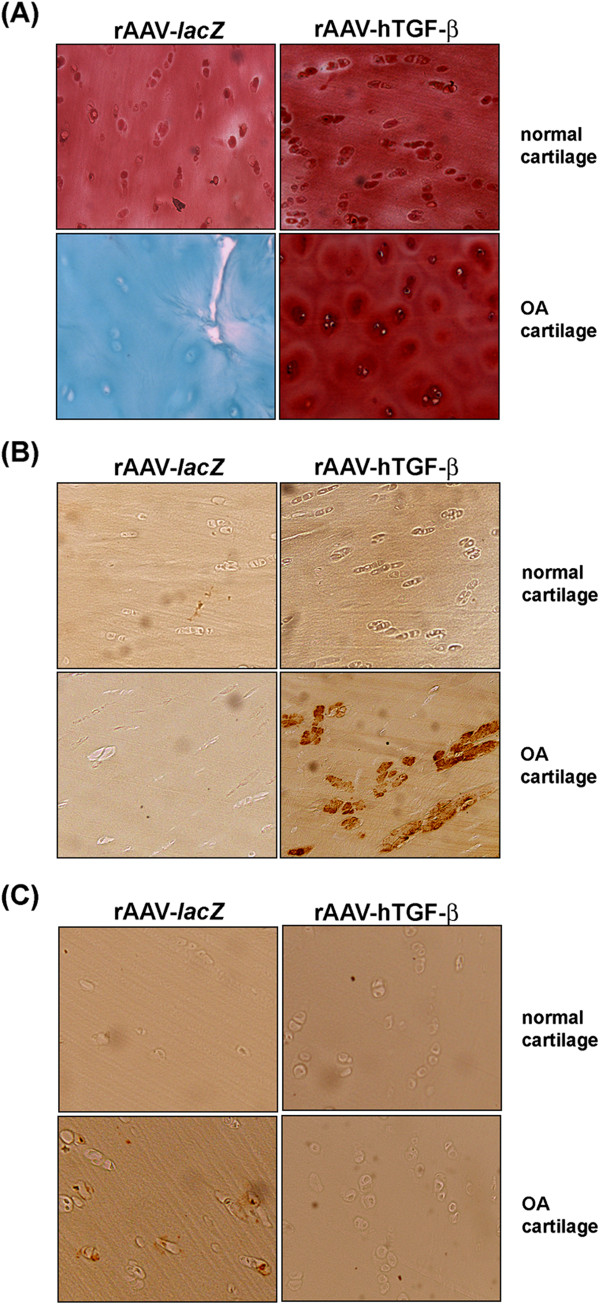
**Effects of rAAV-mediated TGF-β expression upon the anabolic activities of human normal and OA chondrocytes *in situ*.** Explants were transduced with rAAV-*lacZ* or rAAV-hTGF-β as described in Figure 1 and maintained in culture for 90 days. The samples were then fixed and histologically processed for safranin O staining **(A)** and immunohistochemical detection of type-II **(B)** and type-X collagen **(C)** (all at magnification x10; view of the middle zone).

*In vitro*, immunodetection of BrdU incorporation revealed significant and durable (from day 5 to day 21) increases in the levels of cell proliferation with TGF-β *versus lacZ* both in normal and OA cells (up to 6.3-fold difference, always *P* ≤ 0.001) (upper panels of Figures 2A and 3A). These results were corroborated by Cell Proliferation ELISA BrdU (0.698 *versus* 0.605 OD^450 nm^ in normal cells and 0.680 *versus* 0.626 OD^450 nm^ in OA cells; up to 1.2-fold difference, always *P* ≤ 0.001) and by analyzing the DNA contents (up to 1.3-fold difference, always *P* ≤ 0.001) (Figure 3B). Similar results were noted in cartilage explant cultures *in situ*. Immunodetection of BrdU incorporation in normal and OA explants demonstrated significant and durable (from day 21 to day 90) increases in the levels of cell proliferation with TGF-β *versus lacZ* (up to 15.8-fold difference, always *P* ≤ 0.001) (lower panels of Figures 2A and 4A). These findings were substantiated by an analysis of the DNA contents (up to 2.3-fold difference, always *P* ≤ 0.001) (Figure 4B) and of the cell densities on histological sections (up to 4.7-fold difference, always *P* ≤ 0.001) (Figures 4C and 5A). Remarkably, these parameters were always higher with TGF-β in normal cartilage *versus lacZ* (always *P* ≤ 0.001). Of further note, a TUNEL analysis showed that the presence of TGF-β significantly and durably (from day 21 to day 90) reduced the percentage of apoptotic cells in OA cartilage compared with *lacZ* (36-fold decrease, *P* ≤ 0.001), bringing back the levels to those noted in control normal cartilage (almost undetectable levels) (Figures 2B and 4D).

Further biochemical analyses *in vitro* next revealed significant and durable (from day 5 to day 21) increases in the proteoglycan and type-II collagen contents with TGF-β *versus lacZ* both in normal and OA cells (up to 11.5-fold difference, always *P* ≤ 0.001) (Figures 3C and D) while those for type-X collagen significantly and durably decreased (from day 5 to day 21) with TGF-β (up to 1.7-fold difference, *P* ≤ 0.001 in OA cells) (Figure 3E). Again, similar results were obtained in cartilage explant cultures *in situ*. An analysis of the proteoglycan and type-II collagen contents showed significant and durable (from day 21 to day 90) increases with TGF-β *versus lacZ* both in normal and OA cartilage (up to 8.2-fold difference, always *P* ≤ 0.001) (Figures 4E and G). These findings were substantiated by an analysis of the intensities of safranin O staining and of type-II collagen immunostaining (up to 17.4-fold difference, always *P* ≤ 0.001) (Figures 4F,H, 5A, and B). Again, these parameters were always higher with TGF-β in normal cartilage *versus lacZ* (always *P* ≤ 0.001). Also, the contents and immunostaining intensities for type-X collagen significantly and durably (from day 21 to day 90) decreased with TGF-β (up to 20.5-fold difference, *P* ≤ 0.001 in OA cartilage) (Figures 4I, J, and 5C).

These findings show that application of rAAV-hTGF-β is capable of both enhancing the proliferative and anabolic activities of human normal and OA chondrocytes *in vitro* and *in situ* while advantageously delaying their terminal differentiation. While the effects of TGF-β were in general more robust early on both *in vitro* and *in situ* (between 1.1- and 1.7-fold difference), probably due to higher levels of TGF-β expression over time (up to 3.04-fold difference), they remained significant *vis-à-vis lacZ* at the latest time points evaluated (always *P* ≤ 0.001).

### Evaluation of the pathways allowing for the long-term protective effects of TGF-β via rAAV gene transfer in human normal and OA articular cartilage

To determine the mechanisms possibly involved in the processes of TGF-β-mediated cartilage remodeling over time via rAAV gene transfer, we investigated the expression of critical chondrocyte differentiation-related and OA-associated factors in the cartilage *in situ* at the latest time point evaluated in the study (90 days) among which MMP-13 (collagenase-3, a marker of terminal differentiation), the members of the protective TIMP family (TIMP-1 and −3), PTHrP (a hypertrophy-associated agent), β-catenin (a mediator of the Wnt signaling pathway associated with hypertrophy), and the TGF-β receptor I (protective ALK5 signaling pathway *versus* alternative opposing ALK1 route).

**Figure 6:**
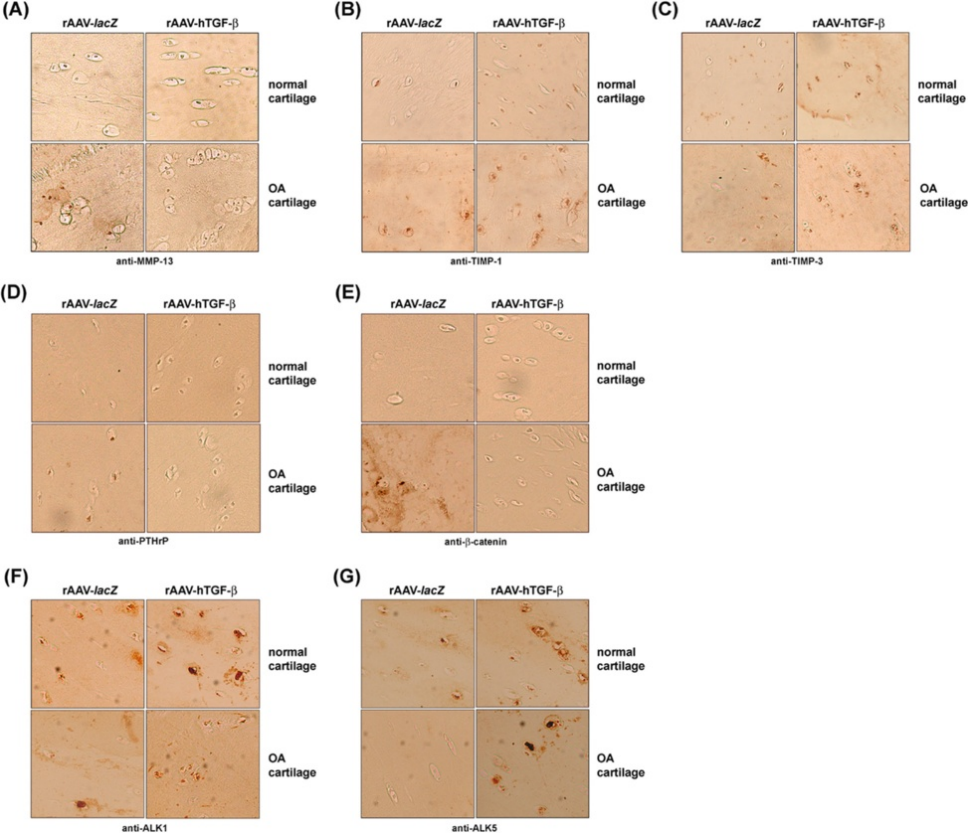
**Potential mechanisms and pathways involved in the effects of TGF-β in rAAV-transduced human normal and OA chondrocytes *in situ*.** Explants were transduced with rAAV-*lacZ* or rAAV-hTGF-β as described in Figure 1 and maintained in culture for 90 days. The samples were then fixed and histologically processed for immunohistochemical detection of MMP-13 **(A)**, TIMP-1 **(B)**, TIMP-3 **(C)**, PTHrP **(D)**, β-catenin **(E)**, and the TGF-β receptor I (ALK1 and ALK5) **(F** and **G**, respectively**)** (all at magnification x20; view of the middle zone).

**Figure 7:**
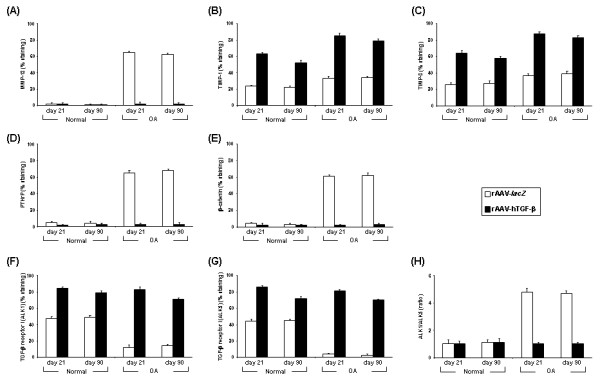
**Morphometric analyses of mechanisms and pathways in rAAV-transduced chondrocytes *in situ*.** Explants were transduced with rAAV-*lacZ* or rAAV-hTGF-β as described in Figure 1 and maintained in culture for up to 90 days. The samples were then fixed and histologically processed at the denoted time points to monitor the % of cells immunostained for MMP-13 **(A)**, TIMP-1 **(B)**, TIMP-3 **(C)**, PTHrP **(D)**, β-catenin **(E)**, and the TGF-β receptor I (ALK1 and ALK5) **(F** and **G**, respectively**)**. Data on the ALK1/ALK5 ratio are presented in **(H)**.

Administration of rAAV-hTGF-β to OA cartilage *versus* rAAV-*lacZ* promoted a significant decrease in the levels of key components involved in hypertrophic differentiation such as MMP-13 (31-fold, *P* ≤ 0.001) (Figures 6A and 7A), PTHrP (22.7-fold, *P* ≤ 0.001) (Figures 6D and 7D), and β-catenin (20.7-fold, *P* ≤ 0.001) (Figures 6E and 7E) while expression of these markers was low in normal cartilage. In contrast, expression of the protective TIMP-1 and TIMP-3 significantly increased following application of TGF-β both in normal and OA cartilage (at least 2.3-fold for TIMP-1 and 2.1-fold for TIMP-3, always *P* ≤ 0.001) (Figures 6B and 7B and Figures 6C and 7C, respectively). As a result, the proportion of TIMPs against MMP-13 was significantly higher in TGF-β- than in *lacZ*-treated (control) OA cartilage (39.5 *versus* 0.5 for TIMP-1/MMP-13, i.e. 79-fold; 41.5 *versus* 0.6 for TIMP-3/MMP-13, i.e. 69.2-fold; always *P* ≤ 0.001) and than in control normal cartilage (22 for TIMP-1/MMP-13, i.e. 1.8-fold; 27 for TIMP-3/MMP-13, i.e. 1.5-fold; always *P* ≤ 0.001). Transduction with rAAV-hTGF-β was also capable of enhancing the expression of the TGF-β receptor I in normal and OA cartilage compared with control conditions. Both the levels of ALK1 and ALK5 were significantly up-regulated in response to TGF-β (ALK1: 1.6-fold in normal and 5.1-fold in OA cartilage; always *P* ≤ 0.001; ALK5: 1.6-fold in normal and 23.3-fold in OA cartilage; always *P* ≤ 0.001) (Figures 6F and 7F and Figures 6G and 7G, respectively). Strikingly, while similar increases were noted for ALK1 and ALK5 in normal cartilage with TGF-β allowing to maintain the ALK1/ALK5 ratio to ~ 1.1 like in the corresponding controls (Figure 7H), application of the therapeutic vector to OA cartilage enhanced the ALK5 levels to those noted for ALK1 thus re-establishing a standard ALK1/ALK5 balance in OA (~ 1.0) *versus* a shift towards increased, unfavorable ALK1 noted in damaged, control cartilage (~ 4.7) (Figure 7H).

These findings indicate that treatment of human OA cartilage with the candidate rAAV TGF-β vector beneficially impacts the processes of chondrocyte hypertrophy and terminal differentiation in human OA chondrocytes *in situ* via the TGF-β signaling pathway.

## Discussion

### Study goals

Direct therapeutic gene transfer based on the use of the efficient and stable rAAV vectors is a promising tool to manage the irreversible progression of OA. In this regard, TGF-β might be a good candidate to achieve this goal due to its protective and reparative effects in the articular cartilage [32,33]. Notably, Ulrich-Vinther *et al*. [34] reported that gene transfer of TGF-β via rAAV was capable of increasing the levels of key ECM components while decreasing those of MMP-3 over a one-week period of time in human OA chondrocytes *in vitro*, yet the benefits of such an approach upon the long-term remodeling of human OA cartilage especially *in situ* remain to be elucidated. In the present study, we therefore examined whether an rAAV-hTGF-β vector can effectively and durably modify primary human normal and OA articular chondrocytes *in vitro* and most importantly in cartilage explant cultures *in situ*, leading to a prolonged activation of remodeling activities compared with control treatment.

### rAAV mediates successful overexpression TGF-β in human normal and OA articular chondrocytes *in vitro* and *in situ*

For the first time to our best knowledge, we show that efficient, sustained TGF-β expression can be promoted by rAAV gene transfer both in human normal and OA chondrocytes *in vitro* for at least 21 days and in human normal and OA cartilage explants *in situ* for at least 90 days, probably resulting from the persistence of rAAV in the targets [24], and with transduction efficiencies reaching 70-80% in these systems, in good agreement with previous findings using this class of vector [24,26,28,34,37,38]. The levels of production achieved here early on *in vitro* with rAAV (up to 552.4 pg TGF-β/ml/24 h on day 5 at an MOI = 40) were in the range of those reported by Ulrich-Vinther *et al*. at a similar time point (5 ng/ml/24 h on day 8 at an MOI = 250) [34]. For comparison, the levels of expression reached 60 ng/ml/24 h with a nonviral vector but in bovine chondrocytes and using a very high amount of plasmid (2 μg) [48], 2.5 ng/ml/24 h with an adenoviral vector at an MOI of 50 but in a human chondrocyte-like cell line [35], and 20–33 ng/10^5^ cells/24 h (i.e. 4–7 ng/2 × 10^4^ cells/24 h) in human chondrocytes with retroviral vectors but tested upon selection of transduced cells [40,41]. However, only very short-term expression was noted with these classes of vectors (never beyond 4 days) while we describe an ongoing, significant synthesis until day 21 (up to 219.4 pg/ml/24 h). Most remarkably, and for the first time, we further evidenced a sustained production of TGF-β *in situ* via rAAV (up to 90 days), reaching levels of up to 987.7 pg/ml/24 h and occurring through the whole thickness of the cartilage, probably due to the ability of the small rAAV particles to penetrate the dense matrix [24,26,28,38].

### rAAV-mediated TGF-β overexpression activates the proliferative and anabolic activities of human normal and OA articular chondrocytes *in vitro* and *in situ*

The data further indicate that such high, maintained levels of rAAV-delivered TGF-β stimulated both the proliferative, survival, and biosynthetic activities of human normal and OA chondrocytes *in vitro* and *in situ* over time compared with control treatments, consistent with the properties of the growth factor [23,34-36,39-41]. A rigorous comparison of the effects of TGF-β resulting from rAAV gene transfer compared with other vector classes is difficult to establish as divergent assessment methods have been used in these earlier studies [23,34-36,39-41]. Nevertheless, it is noteworthy that only short-term effects of the growth factor have been demonstrated there (only some few days) or following cell selection, and mostly in *in vitro* settings, whereas we report prolonged effects both *in vitro* and most significantly *in situ*.

### rAAV-mediated TGF-β overexpression delays chondrocyte hypertrophy and terminal differentiation *in situ* via the TGF-β signaling pathway

Furthermore, application of the current TGF-β construct led to advantageous decreases in the expression of key OA-associated markers of chondrocyte hypertrophic and terminal differentiation like type-X collagen, MMP-13, PTHrP, and β-catenin, again in agreement with the effects of this growth factor [49,50]. In contrast, TGF-β overexpression increased (although to a lesser extent) the levels of protective TIMPs as previously described [51,52], allowing nevertheless to beneficially influence the balance between TIMPs and MMP-13 and suggesting that other pathways might be implicated. Most strikingly, we show that efficient, sustained production of TGF-β via rAAV significantly enhanced the levels of the critical TGF-β receptor I as previously reported [53], both for the ALK1 and ALK5 signaling pathways but in a fashion that restored a favorable, original ALK1/ALK5 balance in OA cartilage like in control normal cartilage [54,55], allowing to overcome the age- and OA-associated changes in TGF-β signaling [56] and probably resulting in the modulation of hypertrophic and terminal differentiation processes [54].

### Perspectives

Interestingly, overexpression of TGF-β in the conditions applied here led to enhanced biological activities in human OA cells and cartilage compared with control normal cells and cartilage. It remains to be seen whether such prominent activities will not alter the cell activities and cartilage and joint integrity over time especially *in vivo*, in light of reports showing adverse effects of TGF-β delivery in experimental animal models (synovial inflammation and fibrosis, osteophyte formation) [57-62]. Still, in these studies, detrimental effects were evidenced when very high amounts of recombinant factor were applied (100–200 ng while we report up to 987.7 pg biologically active TGF-β/ml/24 h with rAAV *in situ*), in a dose-dependent and recurrent manner [62], or following adenoviral-mediated gene transfer at much higher doses than those used here (10^7^-10^9^*versus* 8 × 10^5^ viral particles) [57-61]. It is also important to note that in all these studies, administration of the treatments was performed by intra-articular injection, a setting where the gene vector and recombinant factor can target all the tissues of the joint, allowing TGF-β to possibly exert chemoattractant, inflammatory, and chondrogenic effects especially upon the periosteum, subchondral bone, and synovium [63-65] that is highly permissive to gene transfer [66].

In any case, careful optimization of rAAV TGF-β delivery and expression *in vivo* (dose, duration, localization) will be necessary to establish an effective and appropriate treatment for human OA that takes advantage of the favorable actions of the growth factor over its potentially deleterious effects. Beside injecting low vector doses as performed here, the use of regulatable (tetracycline-sensitive), disease-inducible (NF-κB, COX-2, proinflammatory cytokines), or tissue-specific control elements (SOX9, type-II collagen, cartilage oligomeric matrix protein) may permit to modulate transgene expression compared with the strong CMV-IE promoter. Another important consideration will be to carefully decide on the route of administration. Instead of a conventional approach by intra-articular injection, direct local application of the vector preparation to the sites of cartilage injury might be more favorable to prevent dilution of the treatment in the joint space leading to undesirable dissemination and uptake by surrounding tissues. This will be practicable only when some cartilage surface is remaining like in early stages of OA and transplantation of TGF-β-modified cells might be needed for more advanced cases of the disease, having the further advantages of containing the TGF-β transgene [67] and avoiding transduction of other joint tissues. In this regard, it is interesting to note that Ha *et al*. [68] reported the feasibility of delivering retrovirally TGF-β-modified chondrocytes in patients with severe OA (TissueGene-C dose-escalating phase I clinical trial) with a trend toward efficacy and without serious adverse effects, in marked contrast with findings in experimental systems showing deleterious effects of TGF-β (inflammation, fibrosis, osteophyte formation) when provided at very high and repeated doses [57-62]. Again, rAAV might be best suited to develop such indirect, *ex vivo* trials as their high transduction efficiencies allow to use them without having to preselect the transduced cells compared with retroviral vectors [40,41,67].

Finally, administration of other candidates in conjunction with TGF-β (concomittently or sequentially) might be necessary, especially those that can specifically counteract the side effects of the growth factor or of its putative secondary mediators (fibrotic CTGF, BMP-2 in the case of osteophyte formation) like the inhibitory Smad6 and Smad7 and antagonist gremlin [58,59,69]. Alternatively, agents like IL-1Ra or IL-1 siRNA, sTNFR, NF-κB inhibitors, KBP, TSP-1, DKK-1, POMC, sFlt-1 (a VEGF antagonist) [11,13-21,27] might provide other good options to achieve this goal. Again rAAV might be a powerful tool to achieve these goals as combined gene transfer with this class of vector has been demonstrated in the current systems evaluated [26].

### Final remarks

In summary, the results of the present study indicate that for the first time and in marked contrast with other classes of vectors, the direct, prolonged overexpression of TGF-β via rAAV vectors can efficiently stimulate the reparative activities of human normal and OA chondrocytes over time *in vitro* and most importantly *in situ*, contributing to the significant, proper remodeling of human OA cartilage. Future studies will allow to determine the benefits of applying the rAAV-hTGF-β construct in an appropriate, clinically relevant experimental OA model *in vivo*, requiring to translate first the current findings in the corresponding animal cells. The present findings validate the concept of using rAAV as an effective treatment for human OA.

## Conclusion

OA is an incurable joint disease that disables millions of people worldwide, remaining very difficult to manage. Gene-based approaches may provide long-term treatments to restore an original structure and integrity in OA cartilage by rejuvenating resident (or transplanted) cells. The safe and highly efficient rAAV vectors are particularly well suited to treat OA that is not a life-threatening disease. Here, we showed the potency of an rAAV TGF-β vector to remodel human OA cartilage over extended, clinically relevant periods of time. The effects of this therapeutic vector *in vivo* and upon other affected tissues in the OA joint remain now to be investigated.

## Competing interests

The authors declare that they have no competing interests.

## Authors’ contributions

JKV prepared the vectors and carried out the experiments on gene transfer, cell proliferation and apoptosis, morphometric analyses, and biochemical assays. ARR titrated the vectors and carried out the experiments on transgene expression, histological/immunohistochemical/morphometric analyses, and biochemical assays. GS prepared the cartilage and cell samples for culture and processing and participated in the experiments on morphometric analyses and biochemical assays. AW participated in the experiments on transgene expression, biochemical assays, and statistical analyses. HM participated in the design of the study and helped to draft the manuscript. MC conceived, designed, and coordinated the study, performed the statistical analyses, and wrote the manuscript. All authors read and approved the final manuscript. No writing assistance was used in the production of the manuscript.
